# The Impact of the Circadian Genes *CLOCK* and *ARNTL* on Myocardial Infarction

**DOI:** 10.3390/jcm9020484

**Published:** 2020-02-10

**Authors:** Ivana Škrlec, Jakov Milić, Robert Steiner

**Affiliations:** 1 Histology, Genetics, Cellular, and Molecular Biology Laboratory, Department of Biology and Chemistry, Faculty of Dental Medicine and Health, Josip Juraj Strossmayer University of Osijek, Crkvena 21, HR-31000 Osijek, Croatia; 2 Faculty of Medicine, Josip Juraj Strossmayer University of Osijek, Josipa Huttlera 4, HR-31000 Osijek, Croatia; 3 Clinical Department of Cardiovascular Diseases and Intensive Care, Clinic for Internal Medicine, University Hospital Osijek, Josipa Huttlera 4, HR-31000 Osijek, Croatia

**Keywords:** cardiovascular diseases, circadian rhythm, clock genes, myocardial infarction, polymorphisms

## Abstract

The circadian rhythm regulates various physiological mechanisms, and its disruption can promote many disorders. Disturbance of endogenous circadian rhythms enhances the chance of myocardial infarction (MI), showing that circadian clock genes could have a crucial function in the onset of the disease. This case-control study was performed on 1057 participants. It was hypothesized that the polymorphisms of one nucleotide (SNP) in three circadian clock genes (*CLOCK*, *ARNTL*, and *PER2*) could be associated with MI. Statistically significant differences, estimated by the Chi-square test, were found in the distribution of alleles and genotypes between MI and no-MI groups of the *CLOCK* (rs6811520 and rs13124436) and *ARNTL* (rs3789327 and rs12363415) genes. According to the results of the present study, the polymorphisms in the *CLOCK* and *ARNTL* genes could be related to MI.

## 1. Introduction

Today, there is a global epidemic of cardiovascular diseases (CVDs). The World Health Organization (WHO) data for 2017 shows that CVDs caused 19.9 million deaths globally, and around 80% of CVDs deaths were because of stroke and myocardial infarction (MI) [1]. In recent decades, mortality from CVDs has decreased in developed countries, but CVDs remains one of the principal causes of death worldwide [2]. However, CVDs continue to be the third major cause of death in Croatia, with 45% of total mortality in 2016 [3,4]. Various pathophysiological processes stimulate and lead to the onset of MI. MI is an inflammatory disease. Thrombotic obstruction of the coronary arteries occurs at the position of initiated atherosclerotic plaque. It promotes total coronary circulation arrest, which leads to the death of cardiomyocytes and MI [5]. The etiology of MI is mainly unknown, in spite of the many studies conducted.

The circadian rhythm regulates many physiological mechanisms, and its disruption can result in many physiopathological disorders [6]. The circadian clock is integrated within approximately 24 h [7]. It regulates physiological processes at several levels, from gene transcription to sophisticated performance [8]. Cardiovascular incidents happen in a circadian fashion, with a significant incidence in the morning after rising [9]. Many cardiovascular events show morning circadian preferences, such as myocardial infarction [10], dissection of aortic aneurysms [11], and stroke [12]. Additionally, the incidence of MI is higher during the winter months, especially in the elderly [13]. The circadian rhythm adjusts the feedback of endothelial cells to damage the circulatory system [14]. Some physiological factors that oscillate with the circadian rhythms might trigger the onset of MI [15]. Those physiological factors are glucose homeostasis, blood pressure [16], myocardial contractions, vascular endothelial function, fibrinolytic activity, and metabolism [17,18].

In the peripheral clocks of cardiovascular cells or tissue, the central clock synchronizes and controls the everyday transcription of clock-controlled genes (CCGs) [19]. The primary circadian clock is placed in the SCN (suprachiasmatic nucleus) in the hypothalamus and is controlled by many circadian rhythm genes. Light drives the central clock. Decreased daylight exposure and overexposure to light at night impairs the circadian organization of sleep. Sleep disorders can lead to increased energy input, decreased energy expenditure, and insulin resistance [20]. Short sleep is associated with hypertension, diabetes mellitus, obesity, and mortality. Daylight saving time also causes a modest increase in MI occurrence [21]. Peripheral clocks are found in the cardiomyocyte, blood vessels, and vascular endothelial cells [22]. The circadian clock within cardiomyocytes regulates cardiac metabolic gene expression. It has the function of synchronizing cardiomyocyte metabolic activity with the availability of nutrients [22,23]. Polymorphisms in clock genes are associated with obesity, sleep disturbances, psychological and metabolic complications, plus cardiovascular disorders, such as stroke, vascular death, and myocardial infarction [6]. Desynchronization of the circadian rhythm can cause metabolic disorders and various other issues. Some of those are dyslipidemia, glucose intolerance, hypertension, type 2 diabetes mellitus (T2DM), and CVDs [24,25]. Whole-genome studies have detected numerous genes variants related to the elevated risk of myocardial infarction [26]. Genes included in the metabolic processes of lipid metabolism and the progress of T2DM have been the most investigated genes so far in relation to an enhanced risk of myocardial infarction [7].

Through the transcription and translation feedback loops, circadian rhythm genes control the cyclic transcription of mRNA and protein synthesis. There are some essential proteins in the SCN. The activators are CLOCK (Circadian Locomotor Output Cycles Kaput) and ARNTL (Aryl Hydrocarbon Receptor Nuclear Translocator-Like), while the inhibitors of transcription are PER (Period) and CRY (Cryptochrome) proteins. The day-to-night shift is generated by circadian oscillations that are maintained at the transcriptional and posttranscriptional levels in a single cell by the feedback loop of the circadian clock genes. ARNTL/CLOCK protein heterodimers trigger the expression of PER, CRY, and other clock-controlled genes. CRY/PER protein heterodimers serve as a negative feedback loop and repress the action of ARNTL and CLOCK [8,27]. The entire procedure of activating and suppressing gene transcription in a loop persists for approximately 24 h. Transcription factors stimulate the transcription of clock genes and other clock-controlled genes, initiating numerous physiological processes [7,28].

This research is a continuation of the preliminary study from 2018 [29]. The present study was aimed at searching for a potential correlation between the SNPs of the *CLOCK*, *ARNTL*, and *PER2* genes in patients with myocardial infarction. It was carried out as a case-referent study, comparing participants with myocardial infarction versus the participants without myocardial infarction.

## 2. Experimental Section

### 2.1. Participants

**Figure 1:**
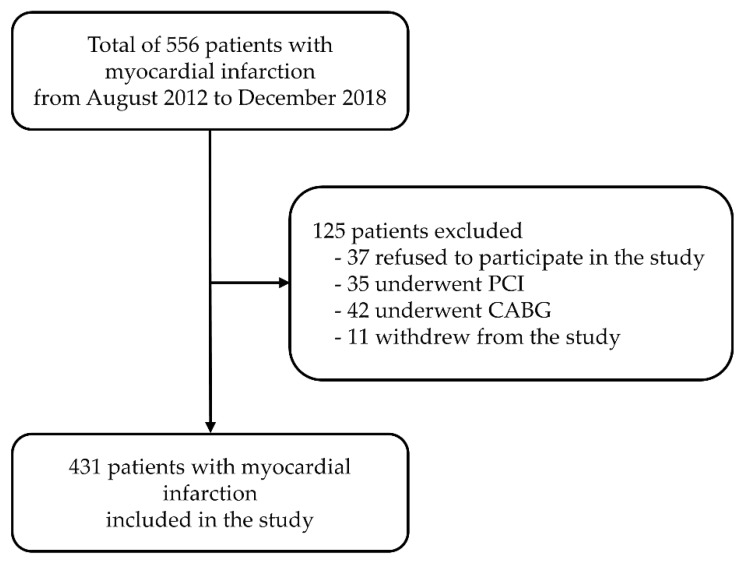
Patient selection flow chart. CABG—coronary artery bypass grafting, PCI—percutaneous coronary intervention.

The data presented here were collected as part of previously reported studies on circadian rhythm variations in patients with myocardial infarction [25,29,30]. The sample included 431 patients (243 males, 188 females) of Croatian origin with non-fatal acute myocardial infarction at the Clinical Department of Cardiovascular Diseases and Intensive Care at the University Hospital Osijek, from August 2012 to December 2018. Inclusion criteria for type 1 and 2 MI patients, according to Thygesen et al., were elevated cardiac troponin T above the 99th percentile and one of the following factors: symptoms of myocardial ischemia, electrocardiogram (ECG) changes, pathological Q waves, evidence of loss of viable myocardium, and coronary thrombus [31,32]. Patients were eliminated from the analysis if they did not satisfy these requirements. Patients were excluded if they had undergone percutaneous coronary intervention or a coronary artery bypass, because those are not clinical characteristics of acute MI. Patients with type 4 and 5 MI were excluded, and also patients with type 1 and 2 MI who underwent PCI (percutaneous coronary intervention). A total of 125 patients were eliminated from the study: 35 due to percutaneous coronary intervention, 42 due to coronary artery bypass, 37 refused to participate, and 11 withdrew from the study (Figure 1).

Participants (total of 626) whose medical records did not present a history of cardiovascular disease were included in the control no-MI group. Their general physician selected them in the outpatient clinic after a checkup. Patients with cardiovascular disease or MI were eliminated. Due to the complex inheritance of cardiovascular risk factors identified in monozygotic twins, the relatives of the patient were eliminated from the no-MI group [33].

Systematic data on their medical history were obtained from all participants. Data on age, smoking, hypertension, and diabetes mellitus were included in the questionnaire. The patient’s medical record confirms all of the above information.

This study was authorized by the Ethics Committees of the University Hospital Osijek (No. 25- 1:3160-3/2012) and the Faculty of Medicine Osijek (No. 2158-61-07-12-2). It was performed following the Declaration of Helsinki and its amendments. All participants signed informed consent.

### 2.2. SNP Selection and Genotyping

SNPs in three circadian rhythm genes, CLOCK, ARNTL, and PER2, were genotyped in this study. SNPs were selected on the basis of the familiar genetic linkage, consistent with HapMap Phase 3 (http://www.hapmap.org). The collection of the most specific polymorphisms for *CLOCK* (rs11932595, rs6811520, and rs13124436), *ARNTL* (rs3789327, rs4757144, and rs12363415), and *PER2* (rs35333999 and rs934945) genes were acquired using the Tagger algorithm accessible within the Haploview software (Haploview, version 4.2) [34]. Additionally, selected circadian rhythm gene SNPs had been associated with cardiovascular risk factors in previous studies [14,16,18].

The extraction of genomic DNA was made from lymphocytes using conventional methods (QIAamp DNA Blood Mini Kit, Qiagen, Hilden, Germany). Genotyping was performed using TaqMan SNP genotyping assays by the real-time PCR method, performed using a 7500 Real-Time PCR System (Applied Biosystems, Foster City, CA, USA). Details of the selected TaqMan probes are shown in Table 1. Allele discrimination analyses were carried out using SDS 7500 Software Version 2.3 (Applied Biosystems, Foster City, CA, USA).

### 2.3. Statistical Analysis

All analyses were performed using SPSS software (version 22.0, SPSS Inc., Chicago, IL, USA) for Windows). Quantitative demographic and clinical variables are presented as mean and standard deviation, while categorical variables are presented as frequency and percentages. Chi-square tests (χ^2^) on contingency tables were applied to analyze allele and genotype frequencies in both groups. By comparing the distribution of genotypes with those expected by the Hardy–Weinberg equilibrium, a further level of quality control of genotyping was accomplished applying the Chi-square goodness-of-fit test. Analyses were performed using SNPStats [35] and the SHEsis web tools [36]. The relationship between genotypes and cardiovascular risk factors were studied applying the Mann–Whitney U test. Logistic regression, for the likelihood of a patient having a manifestation of myocardial infarction, was applied to evaluate the outcome of *CLOCK* (rs11932595, rs6811520, rs13124436), *ARNTL* (rs3789327, rs4757144, rs12363415), and *PER2* (rs35333999 and rs934945) genotypes. Age, hypertension history, diastolic and systolic blood pressure, and BMI were applied as covariates. The Kruskal–Wallis test was applied to establish the association within genotypes and risk factors. When the *p*-value was equal to or less than 0.05, the associations were marked significant. Appling the Benjamini–Hochberg (false detection rate—FDR value) correction method, significant value corrections were made due to the many SNPs studied. *Q*-values less than 0.05 were deemed significant. Pairwise linkage disequilibrium (LD) and haplotypes were calculated applying the SHEsis web tool [37] and Haploview (version 4.2) because the participants in the present study were not related.

## 3. Results

Table 2 shows the prevalence of cardiovascular risk factors in the 1057 study participants. In the present study, the mean age of all participants was 64 ± 10 years, and 53.4% were males. Table 3 presents allele frequencies, while Table 4 shows the genotype distribution of the *CLOCK*, *ARNTL,* and *PER2* SNPs. In the patients’ group, the *ARNTL* gene SNPs rs3789327 and rs12363415, and rs6811520 and rs13124436 of the *CLOCK* gene deviated from the Hardy–Weinberg equilibrium.

Logistic regression was adjusted to evaluate the independent impact of the chosen polymorphism after modification for cardiovascular risk factors. All tested SNPs showed a significant interaction between age, diastolic blood pressure, and the risk of myocardial infarction (*p* < 0.001) (Table 5). Under the dominant genotype model, a considerable difference was for the rs3789327 and rs12363415 SNPs in the *ARNTL* gene (AA + AG versus GG, *p* = 0.003, OR = 1.52 with 95% CI = 1.12–1.95, and AA + AG versus GG, *p* < 0.001, OR = 3.14 with 95% CI = 2.13–4.62, respectively). Under the recessive genotype model notable difference was for the rs6811520 and rs13124436 SNPs in the *CLOCK* gene (CC versus CT + TT, *p* < 0.001, OR = 0.35 with 95% CI = 0.26–0.47, and GG versus AG + AA, *p* = 0.006, OR = 1.76 with 95% CI = 1.17–2.65, respectively). The significant difference was detected under the dominant genotype model for the *PER2* gene polymorphism rs35333999 (TT + CT versus CC, *p* = 0.005, OR = 1.93 with 95% CI = 1.19-3.11).

The association between circadian rhythm gene polymorphisms and cardiovascular risk factors in patients with MI is shown in Table 6.

The completed haplotypes were analyzed in the three circadian rhythm genes. The frequencies of the predicted haplotypes of the tested circadian rhythm gene genetic variants in MI and no-MI participants are presented in Table S1.

There was no linkage disequilibrium (LD) between SNPs in the *ARNTL* gene. The LD between rs3789327 and rs4757144 was D’ = 0.039, $R^{2}$ = 0.001, between rs3789327 and rs12363415 D’ = 0.216, $R^{2}$ = 0.025, and between rs4757144 and rs12363415 D’ = 0.109, $R^{2}$ = 0.005. The LD for the *CLOCK* gene polymorphisms was as follows: the LD between rs1192595 and rs6811520 was D’ = 0.414, $R^{2}$ = 0.108, between rs1192595 and rs13124436 D’ = 0.062, $R^{2}$ = 0.001, and between rs6811520 and rs13124436 D’ = 0.596, $R^{2}$ = 0.103. The LD calculated for polymorphisms in the *PER2* gene (rs35333999 and rs934945) was D’ = 0.986, $R^{2}$ = 0.010 (Figure S1).

## 4. Discussion

In the present study, given the findings of the investigated SNPs of the *CLOCK*, *ARNTL,* and *PER2* genes, it was noted that polymorphisms in those clock genes might be an extra risk factor for myocardial infarction. An association between MI and polymorphisms of the *CLOCK* (rs6811520 and rs13124436) and *ARNTL* (rs37389327 and rs12363415) genes were found in a sample of 1057 participants in this case-referent study. *ARNTL*, rs3789327, was connected with T2DM in MI patients, while rs13124436 and rs6811520 of the *CLOCK* gene were connected with hypertension, T2DM, and systolic blood pressure in MI patients. In the present study, patients with MI had substantially reduced diastolic blood pressure as opposed to the no-MI group. 

The circadian rhythm is a network that enables a person to manage environmental variations and adjust to them. Accordingly, the circadian rhythm controls a range of physiological and metabolic activities, and any obstruction of that rhythm may impact a person’s health. The role of circadian rhythm in MI has been investigated in several studies. Cardiomyocyte circadian gene expression regulates the myocardial contractile purpose, metabolism, and gene expression [38]. The fundamental idea is that acute cardiovascular events do not happen randomly throughout the day but are accelerated partially by circadian controlled factors [39]. An increasing amount of experimental and clinical evidence shows an essential connection between cardiac dysfunction and intrinsic circadian biology. Virtually all tissues of the body have a circadian clock that regulates the timing of many critical biological reactions in response to a diversity of environmental signals. The molecular machinery for the regulation of circadian rhythms is very conserved and found in virtually every cell type [40]. 

Human research has recognized polymorphisms and transcription motifs of circadian rhythm genes, such as *CRY2, CLOCK, ARNTL, PER2*, or *NPAS2,* which are linked with hypertension, metabolic syndrome, or T2DM [30,41]. Circadian rhythm genes play a significant part in homeostatic equilibrium. They adjust the fibrinolytic system, and the *CRY* and *CLOCK* genes are precisely engaged in this action and, accordingly, raise the CVD risk. The purpose of the circadian clock in cardiovascular activity has been strongly emphasized in many studies. However, this research showed an association of MI with some circadian clock polymorphisms. This is relevant because approximately 43% of all protein-coding genes have circadian transcription in an organ-specific way [40]. 

Circadian rhythm genes play a crucial role in many physiological activities. The *ARNTL* gene is an integral part of lipid metabolism due to its influences on the transcription of genes included in lipogenesis in adipocytes in a circadian fashion, as shown in animal models [24]. In humans, *CLOCK* gene SNPs are connected to body weight, metabolic syndrome risk, and insomnia [24,42], while *PER2* and *PER3* gene SNPs are linked to sleep disturbances [43]. Different polymorphisms of circadian clock genes are connected with various cardiovascular disease risk factors [44]. Thus, metabolic syndrome, T2DM, and stroke are associated with *CLOCK* gene variants [29,45,46], while myocardial infarction is associated with *CRY2* and *PER2* gene variations [29]. Metabolic syndrome in humans is associated with the transcription of *CRY1* and *PER2* in adipose tissue [41,47]. Patients with diabetes have more severe atherosclerotic changes in their blood vessels [48] and have a two to three times higher risk of developing cardiovascular disease than healthy subjects [46]. Lifestyle and metabolic disorders are directly related, as indicated by biological and epidemiological studies [49], although the biochemical and genetic connection of the circadian clock with metabolic disorders has not been investigated in detail. Therefore, the significance of the circadian rhythm in the preservation of “energetic” equilibrium and metabolism is obvious. 

Tsai et al. showed the significance of the circadian clock in cardiomyocytes in mice with mutated clock genes. They pointed out that the circadian rhythm is a primary controller of cardiac triglyceride metabolism [50], whereas *ARNTL* gene deletion in adipocyte causes obesity [51]. Polymorphisms of the *BMAL1* gene, which is the mouse equivalent of the human *ARNTL* gene, are connected with hypertension and T2DM [52,53], just as in this study where we found an association of rs3789327 and rs12363415 with T2DM in MI patients. All these data confirm the responsibility of *ARNTL* polymorphisms in the metabolic disorders in humans. The *ARNTL* gene SNPs are associated with hypertension, diabetes mellitus, and metabolic syndrome. All these conditions increase the risk of MI [54]. *BMAL1* was shown to be responsible for the daily variations in blood pressure [40], and here, we found an association of rs12363415 with diastolic and systolic blood pressure. Physiological rhythms of heart rate and blood pressure are lost in *Bmal1* knockout mice [55], and such mice developed dilated cardiomyopathy [56]. *BMAL1* knockout mice had depressed cardiovascular circadian rhythms and developed age-dependent dilated cardiomyopathy. The knockout of *BMAL1* or deletion of all three isoforms of *Cry* in mice contributed to arterial stiffness and the impairment of extracellular matrix composition [40]. 

Genetic variations in the *CLOCK* gene are connected with T2DM and cardiovascular disorders in humans [29,30,41,53,57]. Here, an association of rs6811520 and rs13124436 with T2DM was found in MI patients. Studies using *CLOCK* mutant mice show the crucial function of the *CLOCK* in myocardial contractility, basal metabolism, and daily pulse rate control [41]. Mutation of the *Clock* gene in mouse cardiomyocytes only disrupts circadian rhythm in cardiomyocytes. This *Clock* mutation leads to physiological changes in cardiomyocytes. Some of these are heart rate, cardiac metabolism, response to external signals, contractility, and cardiac growth and regeneration [38]. CLOCK is a transcription factor and one of the primary regulators of the transcription of the circadian clock gene. It is also the regulator of expression of the various transcription factors required for control of the diverse physiological and behavioral processes that occur in a circadian fashion [58]. In this study, rs11932595 SNP of the *CLOCK* gene was connected with systolic blood pressure in MI patients. CLOCK, thrombomodulin, and plasminogen activator-1 inhibitor are required for the endothelial homeostasis. The circadian rhythm regulates genes in vascular endothelial cells [41], and disorder of clock could cause atherosclerosis and MI. Specific *CLOCK* gene polymorphisms are linked to excess weight [59,60], metabolic syndrome, and CVD [61]. A mutation in *CLOCK* leads to the development of age-dependent cardiomyopathy in male mice [40]. 

*PER2* polymorphisms could be connected with myocardial infarction [29]. In this study, the *PER2* genetic variant rs35333999 is connected with age in MI patients. PER2 in the heart plays a crucial part in myocardial ischemia and fatty acid metabolism [62]. In contrast, mutations in the *PER2* gene are connected with a shortened circadian period during constant darkness [63]. In the mouse heart, PER2 has a protective function during myocardial ischemia [62,64]. It is included in the control of fatty acid metabolism with raised utilization of oxygen. In the hearts of mice with clock gene mutations, lipolysis is considerably reduced, which can accelerate the development of metabolic disorders, such as atherosclerosis, which could result in MI [50]. *PER2* knockout mice had more extensive infarct sizes, and the *PER2* in the heart exhibits an essential function during inflammation in myocardial ischemia and reperfusion [64]. The reduction of glycogen storage leads to enlarged infarct areas in mice with *PER2* mutation as a result of lowered glycolysis during myocardial ischemia. Suarez-Barrientos et al. observed that the infarct area was more extensive early in the morning [61], which is similar to the conclusion of Eckle et al. that PER2 stabilization dependent on light had a cardioprotective function in ischemia [65]. The deletion of the *Per2* gene in mice reduces the severity of MI because it limits inflammatory processes, reduces apoptosis, and promotes cardiomyocyte hypertrophy. Therefore, *Per2* gene disruption has a protective function in MI [66]. Activation of PER2 at the time of ischemia controls the beta-oxidation of fatty acids and inflammatory processes. Inflammation and metabolism are related, and inflammation might be an outcome of a disturbed metabolism [61]. Patients with raised inflammatory markers and metabolic syndrome have a higher chance of developing CVD. Disturbance of the circadian rhythm is involved in the development of a CVD, and hypertension is an essential element [30,53]. Furthermore, a mutation in the *PER2* gene was connected with aortic endothelial dysfunction, it reduces the production of nitric oxide, and other vasodilatory prostaglandins [40]. Per1 was also found to control the expression of many genes linked to sodium transport in the kidneys [40]. 

Genetic factors may explain, at least in part, some susceptibility to MI in individuals exposed to chronic or even short-term disorders. Thus, shift work is associated with a small but significant increase in the risk of developing CVD, which means that sleep disorders make an essential contribution to the risk of developing CVD [67]. Shift work and physical inactivity are associated with CVD risk factors such as increased triglyceride levels, which can lead to increased risk for MI [68]. A family history of CVD is another risk factor for developing CVD, especially premature CVD, and sudden cardiac death. In addition to genetic factors, a lifestyle passed on from generation to generation has a significant impact on the development of CVD. The increased genetic predisposition for MI, along with inherited life patterns of physical inactivity, may explain to some extent the increase in the incidence of MI [69]. Studies have shown an association between daylight saving time and the incidence of myocardial infarction. A particularly significant increase in the incidence of MI was observed in the first week after the spring shift [21]. The shift to daylight saving time causes circadian rhythm disturbance and sleep disruption, thereby increasing the risk of MI. Therefore, the abolition of daylight saving time is considered to be good for human health and might reduce the risk of CVD [70].

Cardiovascular disease and metabolic disorders are associated with circadian rhythm disturbances in humans [71]. It is crucial to know the ethnic structure of the investigated population since the frequency of circadian rhythm gene polymorphisms differs significantly between different populations [72]. This study investigated the relationship between myocardial infarction patients and various circadian clock genes polymorphisms. The present study examined the prevalence of genetic variants and several genotype models within two groups, patients with myocardial infarction and no-MI participants. Specific genetic variants of circadian rhythm genes might affect the ability of individuals to adapt their circadian rhythm to changing environmental conditions. The results of the present study are compatible with new presumptions, where circadian rhythm aberrations play an essential part in the onset of MI. 

A possible explanation for the lack of correlation of all the studied polymorphisms of the circadian rhythm genes with MI is that individual SNPs have a minor input in the onset of MI. Only when certain combinations of specific SNPs are considered together, a significant association might be obtained. It is significant because the circadian rhythm genes are closely connected in a complex clock mechanism. As MI is a complex trait, the humble contributions of numerous genes affect the phenotypic presentation.

### Limitations of the Study

The sample size is a limitation of this research. A more significant association between phenotype and genotype might be obtained with more patients involved. The number of participants was limited and might produce a false-positive result. The inadequate statistical capability to distinguish positive relationships is a result of the small frequency of some genotypes, as well as the excessive ORs and an extensive range of 95% CI. There is a chance of developing some of the CVDs in the no-MI group. A limitation of the study might be the age discrepancy between the MI patients and the no-MI group. However, a significant association only exists for age and one SNP in the *PER2* gene, rs35333999, in MI patients. Moreover, it is plausible that the *CLOCK*, *ARNTL*, and *PER2* gene SNPs studied are not usefully associated with MI. In the present case, useful polymorphisms or a collection of usefully relevant SNPs that could be better related to MI should be identified. This strategy of approaching the MI problem produces the chance to proceed with the investigation in multicenter studies with more participants. An advantage of the sample in this research is that it was nearly homogeneous in demographic variables such as sex, ethnicity, and cultural background.

## 5. Conclusions

The physiological activities of the individual are coordinated daily by the circadian rhythm. The circadian rhythm plays a significant part in numerous features of normal cardiovascular homeostasis and disease pathogenesis and pathophysiology. Circadian clock gene SNPs might be valuable in assessing the risk, prediction, or evaluating the feedback to therapy of CVD patients. Data are presented here indicating that the *ARNTL* and *CLOCK* gene polymorphisms could be associated with MI and therefore suggest the involvement of the circadian clock in the development of MI. These findings might increase knowledge of the physiopathological processes implicated in MI, and need replication in different individuals with MI. Additional confirmation and systematic study of the circadian rhythm in MI are achievable. In this age of personalized medicine, understanding of the genetic background of the circadian rhythm of a person could be essential for therapy and should be incorporated into diagnostic procedures.

## Figures and Tables

**Table 1:** Selected TaqMan probe details.

Gene	SNP	SNP ID	Location	SNP Type
*ARNTL*	rs3789327	C_2160503_20	Chromosome 11	intronic region
*ARNTL*	rs4757144	C_1870683_20	Chromosome 11	intronic region
*ARNTL*	rs12363415	C_31248677_10	Chromosome 11	intronic region
*CLOCK*	rs11932595	C_296556_10	Chromosome 4	intronic region
*CLOCK*	rs6811520	C_31137409_30	Chromosome 4	intronic region
*CLOCK*	rs13124436	C_11821304_10	Chromosome 4	intronic region
*PER2*	rs35333999	C_25992030_10	Chromosome 2	Coding (Iso/Val)
*PER2*	rs934945	C_8740718_20	Chromosome 2	Coding (Glu/Gly)

**Table 2:** Prevalence of cardiovascular risk factors of myocardial infarction (MI) and no-MI participants.

	MI	no-MI	*p*-Value *
Number	431	626	
Male sex (%)	243 (56.4%)	321 (51.3%)	0.102
Age (years)	66 ±11	62 ± 10	**<0.001**
History of hypertension (%)	351 (73.1%)	408 (65.2%)	**0.007**
Smokers (%)	119 (27.6%)	193 (30.8%)	**<0.001**
History of type 2 diabetes mellitus (%)	275 (63.8%)	426 (68.1%)	0.151
Diastolic blood pressure (mm Hg)	79.12 ± 11.39	83.46 ± 11.13	**<0.001**
Systolic blood pressure (mm Hg)	140.04 ± 22.20	142.3 ± 21.42	0.085
BMI (kg/m^2^)	29.78 ± 4.46	29.37 ± 4.59	0.198

BMI, body mass index. *Mann–Whitney U test *p*-value.

**Table 3:** Allele frequencies of the *ARNTL*, *CLOCK*, and *PER2* polymorphisms (N = 1057).

Gene	SNP	Minor Allele	MAF MI	MAF no-MI	*p*-Value	*q*-Value
*ARNTL*	rs3789327 *	G	0.425	0.518	**2.27 × 10^−5^**	**6.05 × 10^−5^**
	rs4757144	G	0.420	0.442	0.324	0.432
	rs12363415 *	A	0.413	0.338	**4.37 × 10^−4^**	**8.74 × 10^−4^**
*CLOCK*	rs11932595	G	0.428	0.437	0.685	0.782
	rs6811520 *	C	0.444	0.620	**7.11 × 10^−15^**	**5.68 × 10^−14^**
	rs13124436 *	A	0.200	0.301	**1.72 × 10^−7^**	**6.88 × 10^−7^**
*PER2*	rs35333999	T	0.038	0.055	0.077	0.123
	rs934945	T	0.172	0.174	0.885	0.885

MAF, minor allele frequency; *q*-value, corrected significant *p*-value by the Benjamini–Hochberg method. * Deviation from the Hardy–Weinberg equilibrium in the MI group.

**Table 4:** Genotype distribution and frequencies of the *ARNTL*, *CLOCK*, and *PER2* polymorphisms.

Gene	SNP	Genotype	Genotype Frequency, N (%)				
			MI	no-MI	*p*-value	$\chi^{2}$	*q*-Value
*ARNTL*	rs3789327 *	AA	161 (37.4%)	164 (26.3%)	**2.7 × 10^−4^**	16.46	**7.2 × 10^−4^**
		AG	174 (40.4%)	237 (43.8%)			
		GG	96 (22.3%)	187 (30%)			
	rs4757144	AA	153 (35.5%)	199 (31.8%)	0.456	1.57	0.608
		AG	194 (45%)	300 (48%)			
		GG	84 (19.5%)	126 (20.2%)			
	rs12363415 *	AA	142 (32.9%)	151 (24.1%)	**0.007**	9.95	**0.014**
		AG	72 (16.7%)	121 (19.3%)			
		GG	217 (50.3%)	354 (56.5%)			
*CLOCK*	rs11932595	AA	142 (33%)	191 (30.6%)	0.589	1.06	0.673
		AG	208 (48.4%)	322 (51.5%)			
		GG	80 (18.6%)	112 (17.9%)			
	rs6811520 *	CC	123 (28.5%)	255 (40.8%)	**3.44 × 10^−15^**	69.33	**2.75 × 10^−14^**
		CT	137 (31.8%)	265 (42.4%)			
		TT	171 (39.7%)	105 (16.8%)			
	rs13124436 *	AA	39 (9%)	123 (19.6%)	**1.26 × 10^−5^**	22.61	**5.04 × 10^−5^**
		AG	94 (21.8%)	131 (20.9%)			
		GG	298 (69.1%)	372 (59.4%)			
*PER2*	rs35333999	CC	399 (92.8%)	560 (89.6%)	0.208	3.14	0.333
		CT	29 (6.7%)	61 (9.8%)			
		TT	2 (0.5%)	4 (0.6%)			
	rs934945	CC	296 (68.7%)	427 (68.2%)	0.986	0.03	0.986
		CT	122 (28.3%)	180 (28.8%)			
		TT	13 (3%)	19 (3%)			

$\chi^{2},$ Chi-square; *q*-value, corrected significant *p* value by the Benjamini–Hochberg method. * Deviation from the Hardy–Weinberg equilibrium in the MI group.

**Table 5:** Odds ratios for myocardial infarction adjusted for cardiovascular risk factors included in the logistic regression model.

Risk Factor	OR (95% CI)	*p*-Value
Age	0.96 (0.95–0.97)	**<0.001**
History of hypertension	1.23 (0.91–1.68)	0.182
Diastolic blood pressure	1.36 (1.02–1.05)	**<0.001**
Systolic blood pressure	1.01 (0.99–1.01)	0.217
BMI	0.98 (0.95–1.07)	0.114

OR, odds ratio; CI, confidence interval.

**Table 6:** The association between cardiovascular risk factors and circadian rhythm gene polymorphisms in patients with MI.

Gene	Sex	Age	History of Hypertension	Smoking	History of T2DM	Diastolic Blood Pressure	Systolic Blood Pressure	BMI
*ARNTL*								
rs3789327	**0.045**	0.953	0.247	0.133	**0.006**	0.309	0.113	0.561
rs4757144	0.844	0.818	0.319	0.273	0.537	0.129	0.121	0.777
rs12363415	0.071	0.872	**<0.001**	0.168	**<0.001**	**0.005**	**<0.001**	**<0.001**
*CLOCK*								
rs11932595	0.177	0.487	0.263	0.319	0.118	0.266	**0.029**	0.229
rs6811520	**<0.001**	0.641	**<0.001**	0.950	**<0.001**	0.820	**<0.001**	0.200
rs13124436	**0.003**	0.665	**<0.001**	0.309	**<0.001**	0.402	**<0.001**	0.028
*PER2*								
rs35333999	0.956	**0.002**	0.720	0.422	0.102	0.521	0.934	0.772
rs934945	0.156	0.104	0.085	**0.048**	0.518	0.734	0.589	0.302

Kruskal–Wallis test *p*-values. T2DM, type 2 diabetes mellitus; BMI, body mass index.

## Supplementary Materials

The following are available online at https://www.mdpi.com/2077-0383/9/2/484/s1, Figure S1: Linkage disequilibrium (LD) analysis among single nucleotide polymorphisms (SNPs) within the same chromosome and gene, Table S1: Frequencies and distribution of probable haplotypes in the MI and no-MI groups.
