# Supplementary material for: The Impact of the Circadian Genes CLOCK and ARNTL on Myocardial Infarction
Source: J Clin Med. 2020 Feb 10;9(2):484. doi: 10.3390/jcm9020484 (PMC7074039; doi:10.3390/jcm9020484)
Supplement: Supplementary file 1 [file jcm-09-00484-s001.zip › jcm-701226-supplementary.docx]

**Table 1.** Frequencies and distribution of probable haplotypes in the MI and no-MI groups.

| **Gene** | **rs3789327** | **rs4757144** | | **rs12363415** | **Frequency cases** | **Frequency controls** | ***p*–value** |
| --- | --- | --- | --- | --- | --- | --- | --- |
| *ARNTL* | A | A | | A | 0.181 | 0.123 | **1.87 x 10^−4^** |
| *ARNTL* | A | G | | G | 0.161 | 0.111 | **8.58 x 10^−4^** |
| *ARNTL* | G | A | | G | 0.157 | 0.200 | **0.012** |
| *ARNTL* | G | G | | G | 0.113 | 0.189 | **2.92 x 10^−6^** |
| *ARNTL* | A | G | | A | 0.078 | 0.088 | 0.419 |
| *CLOCK* | rs11932595 | rs6811520 | | rs13124436 |  | | |
| *CLOCK* | A | T | | A | 0.017 | 0.047 | **2.12 x 10^−4^** |
| *CLOCK* | A | T | | G | 0.350 | 0.278 | **3.71 x 10^−4^** |
| *CLOCK* | G | C | | A | 0.064 | 0.109 | **4.36 x 10^−4^** |
| *CLOCK* | G | C | | G | 0.175 | 0.274 | **1.74 x 10^−7^** |
| *CLOCK* | G | T | | G | 0.181 | 0.041 | **3.44 x 1^−26^** |
| *PER2* | rs35333999 | | rs934945 | |  |  |  |
| *PER2* | C | | C | | 0.790 | 0.772 | 0.334 |
| *PER2* | C | | T | | 0.172 | 0.173 | 0.941 |
| *PER2* | T | | C | | 0.038 | 0.055 | 0.081 |
