# Supplementary figures and images for: The Impact of the Circadian Genes CLOCK and ARNTL on Myocardial Infarction
Source: J Clin Med. 2020 Feb 10;9(2):484. doi: 10.3390/jcm9020484 (PMC7074039; doi:10.3390/jcm9020484)

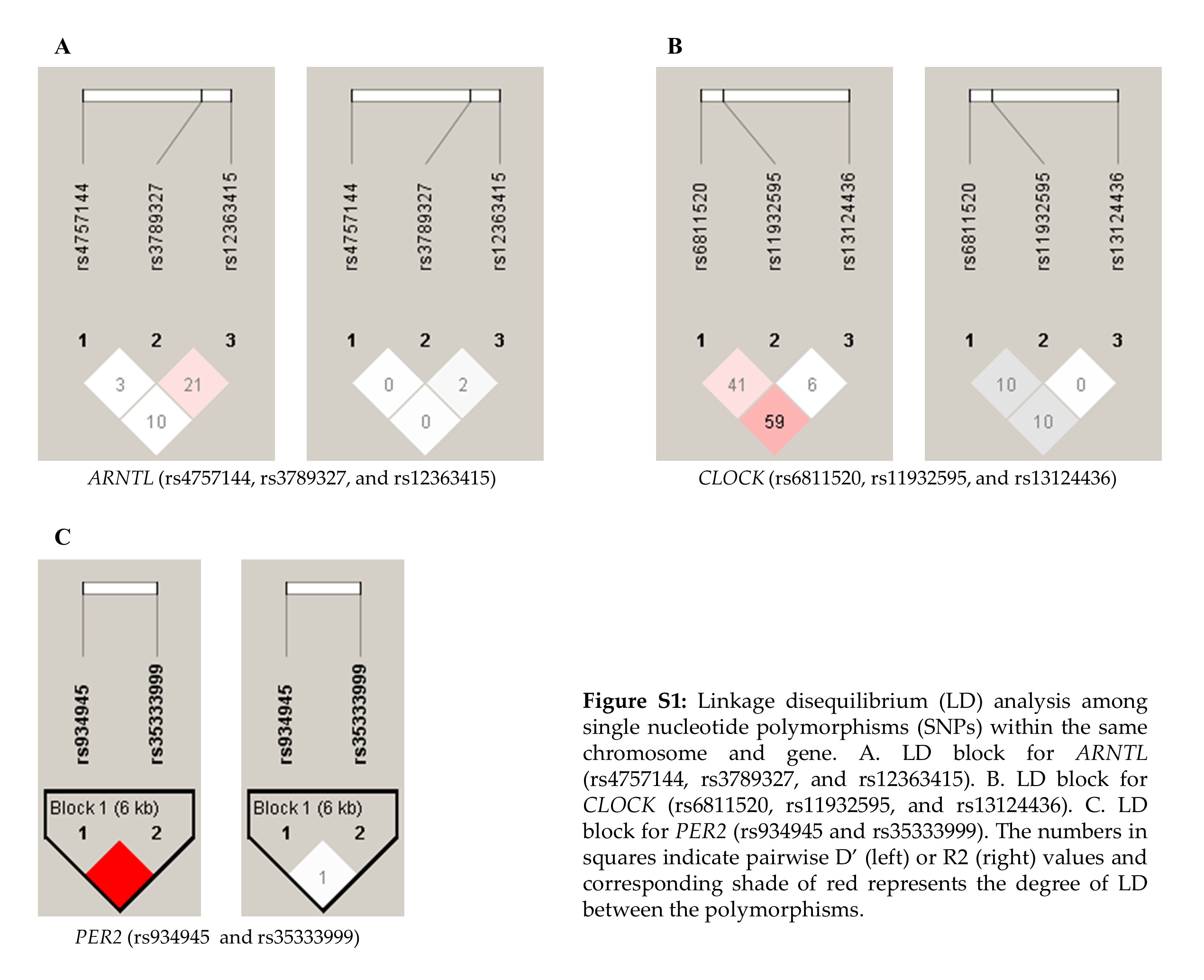

Supplement: Supplementary file 1 [file jcm-09-00484-s001.zip › jcm-701226-supplementaryFigure1.tif]
